# Assessment of susceptible chemical modification sites of trastuzumab and endogenous human immunoglobulins at physiological conditions

**DOI:** 10.1038/s42003-018-0032-8

**Published:** 2018-04-05

**Authors:** Ingrid Schmid, Lea Bonnington, Monika Gerl, Katrin Bomans, Anna Louisa Thaller, Katharina Wagner, Tilman Schlothauer, Roberto Falkenstein, Boris Zimmermann, Jürgen Kopitz, Max Hasmann, Frieder Bauss, Markus Haberger, Dietmar Reusch, Patrick Bulau

**Affiliations:** 1 Pharma Technical Development, Roche Diagnostics GmbH, Nonnenwald 2, Penzberg, 82377 Germany; 2 Pharma Research and Early Development, Roche Diagnostics GmbH, Nonnenwald 2, Penzberg, 82377 Germany; 3 Department of Applied Tumor Biology, Institute of Pathology, Medical School of the Ruprecht-Karls-University, Im Neuenheimer Feld 224, Heidelberg, 69120 Germany

## Abstract

The quality control testing of chemical degradations in the bio-pharmaceutical industry is currently under controversial debate. Here we have systematically applied in vitro and in vivo stress conditions to investigate the influence of protein degradation on structure-function. Extensive purification and characterization enabled identification and functional assessment of the physiological degradation of chemical modification sites in the variable complementarity-determining regions (CDRs) and conserved region of trastuzumab. We demonstrate that the degradation of the solvent-accessible residues located in the CDR and the conserved fragment crystallizable region (Fc) occurs faster in vivo (within days) compared to the levels observed for bio-process and real-time storage conditions. These results hence question the rationality of extreme monitoring of low level alterations in such chemical modifications as critical patient safety parameters in product quality control testing, given that these modifications merely mirror the natural/physiological aging process of endogenous antibodies.

## Abstract (web summary)

Ingrid Schmid and colleagues identified and evaluated the physiological degradation of chemical modification sites of trastuzumab. This study suggests that in vitro PBS incubation studies can be used to predict the protein degradation sites in vivo for critical quality attribute assessment.

## Introduction

Chemical modifications, including asparagine (Asn) deamidation, aspartate (Asp) isomerization and methionine/tryptophan (Met/Trp) oxidation, that occur in proteins have been extensively investigated. Research into the chemical mechanism^1–8^, bioanalytical method development^9–13^, formulation stability and forced degradation^14,15^, and biological impact assessments have been reported^16–18^. Recombinant monoclonal antibodies (mAbs) are exposed to bio-process and storage conditions that can potentially influence the rate and extent of formation of these modifications^19^. Previous studies have shown that degradation of Asn and Asp residues in proteins can affect in vitro stability and in vivo biological function^20–24^. Several IgG1 mAbs have been reported to lose activity as a result of deamidation or isomerization in the complementary-determining regions (CDRs) of the heavy chain^25–30^. In the case of the recombinant IgG1 antibody trastuzumab (Herceptin®), the loss of its potency was reported to be caused by the isomerization of heavy chain Asp-102 (CDR3). On the other hand, the deamidation of the light chain Asn-30 (CDR1) only moderately affects trastuzumab potency^25^. Two studies of other IgG1s reported the heavy chain Asn-55 (CDR 2) to be susceptible to deamidation in vivo^26^ and to exist in a stable succinimide form at mildly acidic pH^27^. In other studies that used different antibodies, the light chain Asp-32 (CDR1), the light chain Asn-33 (CDR1), the light chain Asp-56 (CDR 2), the heavy chain Asp-74, the light chain Asn-92, and the heavy chain Asp-99/101 (CDR3) were found to form succinimide (Asu) or isoAsp^28–31^. Chelius et al.^32^ even applied accelerated degradation conditions to identify four potential deamidation sites in the conserved regions of recombinant IgG1 mAbs.

Oxidation of Met residues in the constant domains of recombinant IgG1 antibodies has been demonstrated to affect the interaction with Protein A, the neonatal Fc receptor and binding to the Fcγ receptors^33–35^. Recently, a clear effect of Met oxidation in the constant region of an IgG1 on the pharmacokinetics has been reported in two in vivo studies^36,37^. So far, however, only one susceptible Met residue within a CDR of recombinant IgG1 antibodies has been reported^30^. In the case of trastuzumab, the heavy chain Met-107 (CDR3) was reported not to be prone to oxidation^38^. Induction of Trp oxidation in the CDRs (heavy chain Trp-105; CDR3) of a mAb by photooxidation resulted in a progressive loss of target binding and biological activity^39^. In another investigation, the light chain Trp-32 (CDR1) of a recombinant IgG1 was found to be susceptible to oxidation under real-time storage and elevated temperature conditions^40^.

In this study, we identified and evaluated the physiological degradation of chemical modification sites of trastuzumab. Our findings suggest that in vitro PBS incubation studies can be used to predict the protein degradation sites in vivo for critical quality attribute assessment.

## Results

### In vitro characterization of trastuzumab degradation sites

**Fig. 1:**
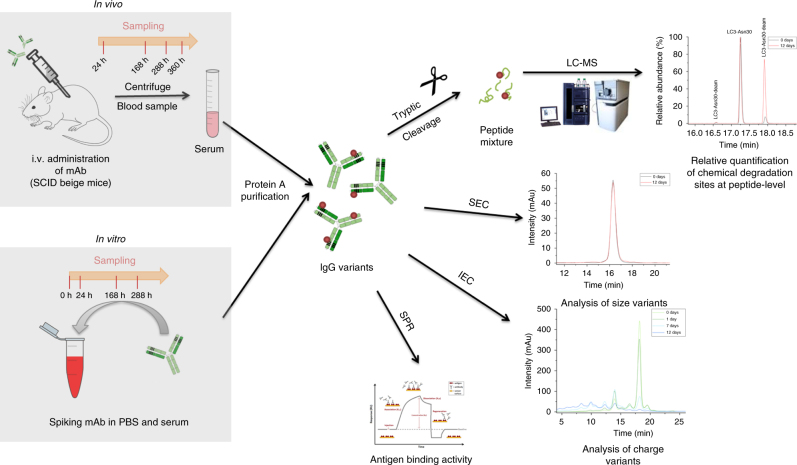
Experimental workflow for the in vitro and in vivo characterization of trastuzumab chemical degradation sites. Application of stress conditions combined with various protein characterization methods enables the identification and functional assessment of the physiological degradation of chemical modification sites in the variable complementarity-determining regions and conserved region of trastuzumab

An approach employing in vitro stress and in vivo conditions was used to assess relevant chemical degradation sites in the CDRs and conserved regions of trastuzumab (Fig. 1).

**Fig. 2:**
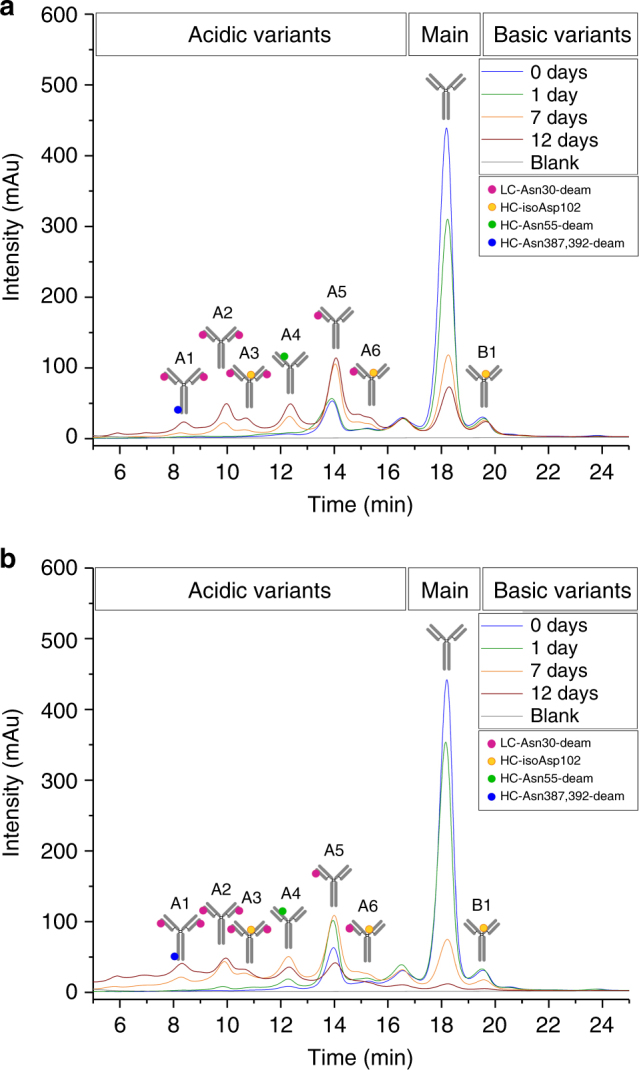
Cation-exchange chromatography of trastuzumab reference material following incubation in **a** PBS and **b** mouse serum. Fractionation and characterization of IgG charge variants was performed by cation-exchange chromatography using a ProPac WCX-10 analytical cation-exchange column (4 × 250 mm) on an UltiMate3000 HPLC system. The trastuzumab charge-variants profile was altered. Increases in the acidic charge variants were observed with a corresponding decrease in native trastuzumab (main peak) but almost no change to the basic charge variants. Analytical characterization data are summarized in Table 1. CEC peak (A1–A6; B1) characterization results are summarized in Table 2. mAU milli-absorbance units

To initially assess trastuzumab degradation sites, we exposed trastuzumab reference material to physiological temperature (37 °C) and pH (7.4) conditions using phosphate buffered saline (PBS) and mouse serum for various incubation periods. Minimal alteration in size variants was observed (fragment and aggregate formation; Supplementary Figures 1A and B) as determined by size-exclusion chromatography (SEC). However, the charge-variants profile, measured by cation-exchange chromatography (CEC) was altered. Increases in the acidic charge variants were observed with a corresponding decrease in native trastuzumab (main peak) but almost no change to the basic charge variants (Fig. 2a, b; Table 1), irrespective of the incubation medium.

**Table 1:** In vitro assessment of trastuzumab degradation sites

Storage duration/temperature	RM/−80 °C	1 d/37 °C/ PBS pH 7.4	7 d/37 °C/ PBS pH 7.4	12 d/37 °C/ PBS pH 7.4	1 d/37 °C/ serum in vitro	7 d/37 °C/ serum in vitro	12 d/37 °C/ serum in vitro
LC–MS peptide mapping [%]							
LC-Asn-30							
LC-deamid-30	8.0 (±0.1)	12.4 (±0.2)	32.2 (±0.2)	45.4 (±0.5)	13.5 (±0.2)	43.4 (±0.7)	66.2 (±0.6)
HC-Asn-55							
HC-deamid-55	2.0 (±0.2)	2.3 (±0.1)	4.5 (±0.1)	6.3 (±0.1)	2.4 (±0.1)	5.9 (±0.2)	10.3 (±0.3)
HC-Asp-102							
HC-isoAsp-102	6.5 (±0.2)	8.2 (±0.3)	13.6 (±0.5)	18.3 (±0.5)	7.3 (±0.2)	13.7 (±0.9)	17.7 (±0.2)
HC-Asn-387,392,393							
HC-deamid-387,392,393	1.0 (±0.1)	1.7 (±0.1)	5.1 (±0.2)	7.3 (±0.2)	1.8 (±0.1)	7.5 (±0.1)	13.6 (±0.5)
CEC							
% Acidic	23.6 (±0.2)	27.1 (±0.5)	62.8 (±0.1)	78.1 (±0.3)	36 (±0.2)	80.2 (±0.5)	96.3 (±0.1)
% Main	69.1 (±0.5)	61.5 (±1.2)	29.3 (±0.5)	16.1 (±0.5)	56.0 (±0.5)	15.9 (±1.4)	2.8 (±0.1)
% Basic	6.3 (±0.1)	6.4 (±0.1)	7.1 (±0.1)	5.7 (±0.1)	6.9 (±0.1)	3.8 (±0.3)	0.9 (±0.1)
SEC							
% Fragments	0.2 (±0.1)	0.2 (±0.1)	0.2 (±0.1)	0.3 (±0.1)	0.2 (±0.1)	1.5 (±0.3)	1.8 (±0.4)
% Monomer	99 (±0.1)	98.9 (±0.2)	98.7 (±0.2)	97.5 (±0.9)	99.1 (±0.2)	96.1 (±0.4)	95.8 (±0.4)
% Aggregates	0.9 (±0.1)	0.9 (±0.1)	1.1 (±0.2)	2.2 (±0.9)	0.7 (±0.1)	2.4 (±0.2)	2.3 (±0.2)
SPR							
% Target binding	100 (±1)	98 (±1)	90 (±1)	84 (±1)	97 (±1)	83 (±1)	72 (±1)

In vitro assessment of trastuzumab degradation sites using temperature/pH degradation conditions (*n* = 3) and quantitative ultra-performance liquid chromatography mass spectrometry (UPLC-MS). The LC–MS peptide mapping relative quantification (reported in %) was conducted by quantification of the selected ion current chromatograms of the modified tryptic peptides relative to the wild type. Trastuzumab charge variants were analyzed by cation-exchange chromatography (CEC). The formation of fragments and aggregates was monitored by size-exclusion chromatography (SEC) and target binding activity was assessed by surface plasmon resonance (SPR) spectroscopy. deamid: total Asp/isoAsp content; RM: reference material stored at −80 °C

The increase in the acidic trastuzumab variants was attributed to extensive Asn deamidation. To verify this the stressed samples were further analyzed by tryptic peptide mapping at pH 6 combined with quantitative ultra-performance liquid chromatography mass spectrometry (UPLC-MS)^28^. Following peptide identification by tandem mass spectrometry, the extent of quantifiable Asn deamidation and Asp isomerization was determined by quantitative evaluation of the modified tryptic peptides relative to their respective unmodified parent peptides as described in the Methods section. The quantification results for those trastuzumab amino acid residues showing alterations in deamidation and isomerization are summarized in Table 1. For light chain LC-Asn-30 (located in the CDR1), the levels of deamidation were found to be greatly increased for both the PBS and mouse serum incubation media (raised from 8 to 45% and to 66%, respectively, after 12 days of incubation), whereas deamidation of heavy chain HC-Asn-55 (located in the CDR 2) was only moderately affected (increased from 2 to 6% and to 10%, respectively). For samples from in vitro serum incubations, we found slightly increased pH values (at around pH 8) after 7 and 12 days of incubation whereas the samples derived from in vitro PBS and in vivo mouse studies remained constant at pH 7.4. Thus, the weaker buffer strength of the serum (in vitro) most likely caused the increased deamidation levels observed for in vitro serum experiments compared to the buffered PBS and in vivo systems (Tables 1, 2, and 3). The HC-Asp-102 (located in the CDR3) also displayed a significant elevation in isoAsp formation (increased from 6 to 18% in both media). For the trastuzumab HC-Asn-387/392/393 motif, located in the conserved region, a moderate increase of Asp/isoAsp formation was observed (up from 1 to 7% and to 14%, respectively). In contrast, no significant Asp isomerization to isoAsp was detected (above 5%) for the two conserved heavy chain Asp residues (HC-Asp-283 and HC-Asp-404). These results are in agreement with previous investigations in which the susceptibility of trastuzumab degradation sites and conserved Asn/Asp residues in recombinant IgG1 antibodies were evaluated^25,28,30,32,41^.

**Table 2:** CEC peak characterization

CEC fraction	A1	A2	A3	A4	A5	A6	Main peak	B1
LC–MS peptide mapping [%]								
LC-Asn- 30								
LC-deamid-30	93.5	94.1	92.5	19.0	49.5	47.6	5.4	4.4
HC-Asn-55								
HC-deamid-55	4.6	4.2	3.7	27.8	1.4	1.1	1.0	1.1
HC-Asp-102								
HC-isoAsp-102	11.2	19.0	44.9	10.1	4.6	38.5	6.0	45.3
HC-Asn-387,392,393								
HC-deamid-387,392,393	29.3	14.5	10.2	1.5	1.6	1.5	1.6	0.9
CEC								
% Purity	78	89	72	76	78	89	72	76
SEC								
% Fragments	<0.1	<0.1	<0.2	<0.1	<0.1	<0.1	<0.1	<0.1
% Monomer	>99	>99	>99	>99	>99	>99	>99	>99
% Aggregates	<0.3	<0.6	<0.8	<0.9	<0.6	<0.6	<0.6	<0.6
SPR								
% Target binding	n.d.	58 (±1)	n.d.	n d.	83 (±1)	80 (±1)	100 (±1)	95 (±1)

Detailed characterization of trastuzumab charge variants by CEC fractionation (according to CEC peak assignment displayed in Fig. 2a) followed by quantitative UPLC-MS tryptic peptide mapping, analytical CEC, SEC and SPR-analysis. The fractions A1–A3 were collected from stressed material (PBS day 12), whereas the fractions A4-B1 were isolated from non-stressed reference material

*A* acidic variant, *B* basic variant, *deamid* total Asp/isoAsp content, *n.d.* not determined

Functional evaluation of the stressed samples using surface plasmon resonance (SPR) technology indicated a correlation between the increase in chemical degradation of LC-Asn-30 and/or HC-Asp-102 and the loss of trastuzumab functionality. Compared to the trastuzumab reference material (activity normalized to 100%) a stepwise reduction to 84% and 72% target binding activity was observed after 12 days of incubation for the PBS and mouse serum, respectively (Table 1).

### Analytical evaluation of trastuzumab charge variants

To assess the impact of LC-Asn-30 and HC-Asp-102 degradation at native protein level in detail, the respective IEC peak fractions (A1–A6; B1) were manually isolated by preparative scale cation-exchange chromatography and extensively characterized using SEC, CEC, UPLC-MS peptide mapping at pH 6, SPR and hydrogen/deuterium exchange-mass spectrometry (HDX-MS) techniques (results summarized in Fig. 2a and Table 2). The resulting SPR data demonstrate that deamidation of LC-Asn-30 moderately impacts the trastuzumab target binding activity compared to the main IEC peak fraction (83% for the acidic/deamidated LC-Asn-30 fraction vs. 100% for the main variant). However, only a minor effect from HC-Asp-102 isomerization on trastuzumab target binding activity was detected (95% for the HC-Asp-102 basic fraction).

**Table 3:** In vivo assessment of trastuzumab degradation sites

Storage duration/temperature	1 d/37 °C/ PBS pH 7.4	7 d/37 °C/ PBS pH 7.4	12 d/37 °C/ PBS pH 7.4	15 d/37 °C/ PBS pH 7.4	1 d/ in vivo	7 d/ in vivo	12 d/ in vivo	15 d/ in vivo
LC–MS peptide mapping [%]								
LC-Asn-30								
LC-deamid-30	11.5 (±0.1)	31.6 (±0.3)	44.1 (±0.6)	50.5 (±0.2)	12.4 (±0.2)	31.7 (±0.1)	43.2 (±0.2)	49.2 (±0.1)
HC-Asn-55								
HC-deamid-55	1.7 (±0.1)	4.0 (±0.1)	5.4 (±0.1)	6.5 (±0.1)	2.0 (±0.1)	4.5 (±0.1)	6.5 (±0.1)	7.5 (±0.1)
HC-Asp-102								
HC-isoAsp-102	6.4 (±0.2)	11.8 (±0.2)	16.1 (±0.5)	18.7 (±0.6)	5.4 (±0.2)	11.2 (±0.1)	15.5 (±0.6)	18.2 (±0.3)
HC-Asn-387,392,393								
HC-deamid-387,392,393	1.5 (±0.1)	4.8 (±0.1)	7.4 (±0.4)	9.1 (±0.3)	1.2 (±0.2)	4.9 (±0.1)	8.2 (±0.5)	10.1 (±0.1)
CEC								
% Acidic variant 1	0.2	2.2	5.3	8.0 (±0.2)	0.3 (±0.1)	2.9 (±0.2)	5.3 (±0.6)	7.3 (±0.4)
% Acidic variant 2	0.4	6.8	11.4	13.3 (±0.2)	1.6 (±0.1)	7.5 (±0.3)	11.5 (±0.2)	14.2 (±0.2)
% Acidic variant 3	0.3	2.7	5.2	7.3 (±0.1)	1.4 (±0.5)	3.2 (±0.2)	5.5 (±0.1)	7.0 (±0.1)
% Acidic variant 4	3.4	9.9	12.6	13.7 (±0.2)	4.3 (±0.6)	11.0 (±0.8)	13.3 (±0.2)	14.4 (±0.1)
% Acidic variant 5	15.9	26.3	26	23.9 (±0.1)	15.5 (±0.2)	26.1 (±0.9)	26.0 (±1.1)	24.9 (±0.5)
% Acidic variant 6	3.6	6.8	8.4	8.6 (±0.1)	3.9 (±0.1)	6.8 (±0.3)	7.6 (±0.1)	7.7 (±0.4)
% Main peak	59.9	29.3	16.7	11.7 (±0.1)	58.1 (±0.2)	29.0 (±1.2)	16.9 (±0.7)	12.0 (±0.2)
% Basic variant 1	6.4	6.7	5.2	4.2 (±0.1)	5.6 (±0.1)	5.8 (±0.1)	4.7 (±0.1)	3.4 (±0.1)
SEC								
% Fragment	0.1 (±0.1)	1.1 (±0.1)	1.5 (±0.1)	1.7 (±0.1)	0.1 (±0.1)	1.6 (±0.1)	1.9 (±0.1)	2.2 (±0.1)
% Monomer	99.2 (±0.1)	97.6 (±0.1)	97.1 (±0.1)	97.0 (±0.1)	95.4 (±0.2)	89.8 (±0.4)	90.8 (±0.3)	90.7 (±0.4)
% Aggregate	0.7 (±0.1)	1.3 (±0.1)	1.4 (±0.1)	1.3 (±0.1)	4.5 (±0.1)	8.6 (±0.4)	7.3 (±0.3)	7.1 (±0.4)
SPR								
% Target binding	100 (±1)	91 (±1)	86 (±1)	84 (±1)	100 (±1)	86 (±1)	81 (±1)	80 (±1)

**Fig. 3:**
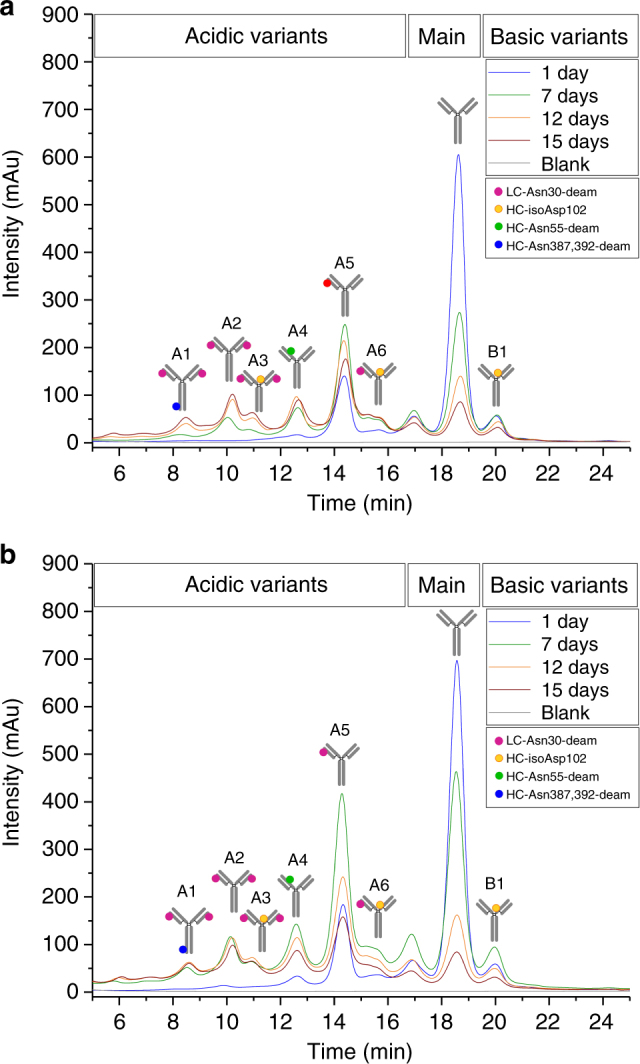
Cation-exchange chromatography of trastuzumab reference material following incubation in **a** PBS and **b** in vivo administration. Characterization of IgG charge variants was performed by cation-exchange chromatography using a ProPac WCX-10 analytical cation-exchange column (4 × 250 mm) on an UltiMate3000 HPLC system. The trastuzumab charge-variants profile was significantly altered. Increases in the acidic charge variants were observed with a corresponding decrease in native trastuzumab (main peak) but almost no change to the basic charge variants. Analytical characterization data are summarized in Table 3. mAU milli-absorbance units

In vivo assessment of trastuzumab degradation sites in SCID beige mouse model for various time points (technical replicates of pooled serum; *n* = ~10 for each time point). CEC variant characterization (A1–A6, Main, B1) is summarized in Fig. 3a. An incubation study with PBS at 37 °C, pH 7.4 was performed in parallel (day 15, *n* = 3; days 1, 7, 12, *n* = 1)

Comparison by bottom-up HDX-MS^42^ over time of the singly deamidated LC-Asn-30 (Fig. 2a; fraction A5) and the singly isomerized HC-Asp-102 (fraction B1) species with native trastuzumab (main peak) showed minimal effect of both chemical modification sites on the trastuzumab higher order structure (see supplementary Figure 2). In detail, no differences in deuterium uptake of the entire variable and conserved trastuzumab sequence were verified (including peptic peptides containing LC-Asn30 and HC-Asp102).

In summary, the results of the range of analyses applied, showed that LC-Asn-30 and HC-Asp-102 represent the most susceptible degradation sites in the CDR of trastuzumab under physiological stress conditions but with low structure-function impact as described above.

### In vivo characterization of trastuzumab degradation sites

Additionally, the in vivo degradation of vulnerable amino acid residues over time was also determined, achieved by administration of trastuzumab in a relevant mouse model and sampling and analysis at selected time intervals. The immune-deficient SCID beige mouse model is commonly used as xenotransplant model and for infectious disease studies (see Methods for details)^43,44^. The low endogenous serum IgG levels of the selected SCID beige mice allow an efficient recovery of administered trastuzumab from serum by Protein A chromatography with minor levels of serum protein impurities (Supplementary Figure 3).

The results from the in vivo administration, recovery and characterization showed minimal alterations in size variants, as determined by SEC (Supplementary Figure 1C). However, the CEC analysis of the Protein A purified mAb showed increases in the acidic variants containing deamidation/isomerization at LC-Asn-30, HC-Asn-55, and HC-Asp-102, with a consequent decrease in the native trastuzumab (main peak; Fig. 3b; Table 3). For the most susceptible deamidation site, LC-Asn-30, greatly increased levels were observed (from 12% up to 49%) after 15 days of administration, as determined by LC–MS peptide mapping. The HC-Asp-102 also exhibited an elevation in isoAsp formation (from 5% up to 18%), whereas deamidation of the HC-Asn-55 was moderately affected (from 2% up to 8%). For the conserved HC-Asn-387/392/393 motive a moderate increase in deamidation was observed (from 1% up to 10%). The results were highly comparable to the data obtained for a parallel study performed with PBS at 37 °C, also provided in Fig. 3a and Table 3.

Functional evaluation by SPR technology demonstrated a correlation between the increase of chemical degradation events in the CDR region and the impact on certain trastuzumab activity, as observed for the in vitro samples. Compared to the trastuzumab reference material (normalized to 100%) a stepwise reduction to 80% target binding activity was observed after 15 days of administration (Table 3).

### Chemical degradation events in the human IgG Fc region

**Fig. 4:**
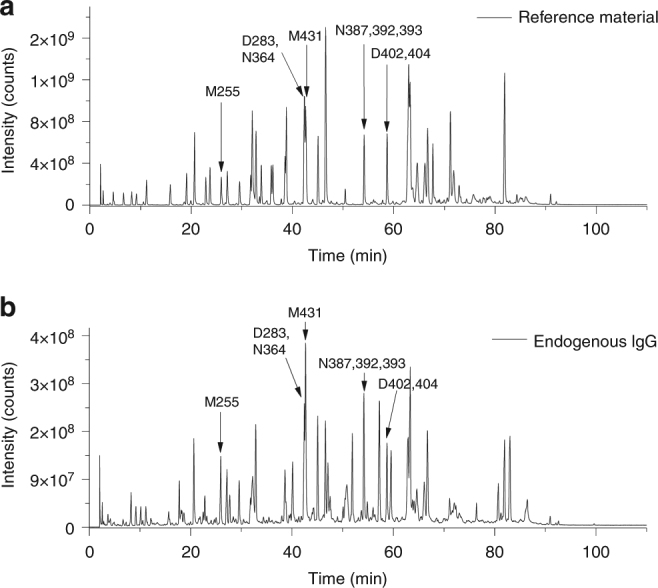
LC/MS total ion current chromatograms of trypsin digested trastuzumab reference material vs. Protein G isolated endogenous human serum IgG pools. Sequence determination of tryptic peptides selected for quantification was achieved by LC–MS/MS. Peptides selected for quantification of Asn (N) deamidation, Asp (D) isomerization, and Met (M) oxidation are marked with arrows. Analytical quantification data are summarized in Table 4

Next we aimed to compare the chemical degradation events in the conserved IgG Fc region of manufactured trastuzumab with the levels found in endogenous human serum IgG. Thus, trastuzumab drug substance reference material (stored at −80 °C for control purposes) and serum IgG’s from healthy human donors were isolated by Protein G chromatography and further analyzed by tryptic peptide mapping at pH 6 as described previously. Predominantly tryptic peptides from the conserved IgG Fc region were observed in the LC–MS total ion current (TIC) chromatogram of the digested serum proteins following Protein G purification suggesting an efficient enrichment of human IgGs from other abundant serum proteins (Fig. 4).

Following peptide identification by tandem mass spectrometry, the extent of quantifiable Asn deamidation, Asp isomerization and also Met oxidation in the conserved Fc region was determined by quantitative evaluation of specific ion current chromatograms (Supplementary Figure 4). The quantification results for those amino acid residues that showed relevant content (≥1%) in deamidation, isomerization, and oxidation are summarized in Table 4.

**Table 4:** Comparison of chemical degradation events in the conserved Fc region

Sample [rel. %]	RM (−80 °C)	RM Control	Endogenous IgG average	Endogenous IgG range (min.–max.)
HC-Asp-283				
HC-Asu-283	3.7 (±0.1)	No difference	3.2 (±0.3)	2.8–3.6
HC-isoAsp-283	0.4 (±0.1)		0.6 (±0.1)	0.4–0.8
HC-Asn-364				
HC-Asu-364	0.3 (±0.2)	No difference	0.2 (<0.1)	0.2–0.2
HC-deamid-364	1.4 (±0.4)		1.0 (±0.1)	0.8–1.3
HC-Asn-387,392,393				
HC-Asu-387,392,393	1.7 (±0.1)	+0.1	1.7 (±0.1)	1.5–2.0
HC-deamid-387,392,393	1.1 (±0.3)		13.7 (±2.5)	8.6–18.6
HC-Asp-402,404				
HC-Asu-404	3.7 (±0.1)	No difference	3.8 (±0.1)	3.6–4.0
HC-isoAsp-404	0.1 (<0.1)		0.3 (<0.1)	0.2–0.3
HC-Met-255				
HC-Met-ox-255	2.3 (±0.2)	+0.4	12.0 (±2.2)	8.1–15.5
HC-Met-431				
HC-Met-ox-431	1.4 (±0.3)	+0.1	7.6 (±1.6)	4.7–9.9

Comparison of chemical degradation events in the conserved Fc region of trastuzumab manufactured with standard cell culture and purification technology with those from endogenous human serum IgG levels (*n* = 15, male/female = 12/3, age = 22–61) as determined by LC–MS peptide mapping. Values reported are percentages relative to the respective wild-type peptide

*Asu* succinimide, *deamid* total Asp/isoAsp content, *RM* reference material, *RM control* reference material purified by Protein G

To assess whether the chemical degradation content of the endogenous human serum IgG’s was altered by the Protein G chromatography purification step applied, trastuzumab drug substance reference material (RM) was also re-processed and re-analyzed by LC–MS peptide mapping. No impact of the Protein G chromatography on trastuzumab degradation was observed (Table 4, RM control).

The identified trastuzumab degradation sites in the conserved Fc region are in agreement with previous studies on IgG1 chemical degradations^28,30,32,38,41^.

For the HC-Asp-283, HC-Asn-364, and HC-Asp-404, we found almost identical deamidation and isomerization levels (1–4% at tryptic peptide level) between the trastuzumab reference material (RM) and endogenous human serum IgG’s. In contrast, the degree of endogenous deamidation at HC-Asn-387/392/393 (up to 20 vs. <2% in trastuzumab RM) and oxidation at HC-Met-255/431 (up to 16 vs. <3% in trastuzumab RM) was higher for human serum IgG’s. In conclusion, chemical degradation of susceptible Fc amino acid residues is elevated in endogenous human IgG pools compared to the trastuzumab reference material.

## Discussion

To identify and evaluate the biological relevance of critical quality attributes that are affected by chemical amino acid modifications in the CDRs and Fc region of mAbs, we took an approach that employs specific in vitro stress conditions. Several studies have evaluated Asn deamidation and Asp isomerization in recombinant antibodies using elevated temperature or other relevant stress conditions^25,27–30,45–49^. In the case of trastuzumab, LC-Asn-30 (CDR1) and HC-Asp-102 (CDR3) have been identified as the most vulnerable degradation sites. The application of elevated temperature stress on the mildly acidic formulated drug substance (pH 6) resulted in the simultaneous and almost equal formation of deamidated LC-Asn-30 and isomerized HC-Asp-102^25,28^. Fractionation of the variants by cation-exchange chromatography was followed by studies regarding functional evaluation of both degradation sites by cell-based anti-proliferation assay^25^. Thus, the use of accelerated temperature conditions with purified and formulated mAb reference material permits an informative preliminary evaluation of the susceptibility of exposed amino acids to chemical degradation under bio-process and storage conditions. The long-term storage stability of therapeutic antibodies (typically at 5 °C and up to 48 months for liquid formulation) is regularly monitored during formulation development and quality control testing of marketed products.

However, only one systematic in vivo assessment of susceptible trastuzumab chemical modification sites (for HC-Asn-55) at physiological conditions (37 °C; pH 7.4) by a validated LC–MS/MS method has been reported so far^50^. In the present study, an approach employing in vitro and in vivo stress conditions combined with multiple analytical techniques for the evaluation of relevant chemical amino acid modifications of trastuzumab in vivo was investigated.

By the in vitro and in vivo application of physiological temperature and pH conditions using PBS, serum and a relevant mouse model, LC-Asn-30 was observed to be the most susceptible trastuzumab degradation site. Specifically, a threefold higher degradation rate compared to the isomerization at HC-Asp-102 and approximately sixfold-elevated deamidation rate compared to the conserved Fc motive HC-Asn-387/392/393 was observed. Moreover, the absolute degradation rate for LC-Asn-30 (~3% per day) was higher compared to a previously published elevated temperature stress (40 °C) study performed under mildly acidic (pH 6) conditions (~1% per day)^28^.

Trastuzumab was subjected to in vivo conditions (pH 7.4, 37 °C) within this study and to accelerated temperature conditions as formulated drug substance (pH 6, 40 °C) in the previous study^28^. In both studies an almost identical degradation/isomerization rate for HC-Asp-102 was observed, demonstrating that the HC-Asp-102 isomerization is predominantly temperature, rather than pH driven. However, a difference in the deamidation of the LC-Asn-30 was verified (threefold increased at pH 7.4 as compared with the pH 6 study) demonstrating that degradation at this site is pH dependent. For HC-Asn-55, a slower degradation rate (0.3% deamidation per day) was verified. The quantitative results are in agreement with a recent study on trastuzumab biotransformation in vivo^50^. In addition, the degradation results for all monitored chemical modifications were highly comparable between PBS incubations and in vivo administrations suggesting that physiological Asn and Asp degradation may be predominantly regulated by pH and temperature and that in vitro PBS incubation studies may facilitate a realistic prediction of protein degradation sites. critical quality attributes assessment exercises based on bio-process and drug storage pH conditions may underestimate the in vivo deamidation of susceptible Asn deamidation sites and should be complemented by adequate stress model systems such as PBS incubations using physiological pH and temperature conditions.

Functional evaluation of in vitro and in vivo stress generated samples by SPR technology demonstrated a comparable correlation between the increase of chemical degradation events in the CDR region and the impact on trastuzumab antigen binding. To explore whether the continuous reduction to 80% target binding activity after 15 days of in vivo administration was related to the elevated LC-Asn-30 deamidation (from ~12% to 50% at reduced peptide level) and/or HC-Asp-102 isomerization (from ~5% to 18%) a detailed analytical characterization of the trastuzumab charge variants following chromatographic fractionation was performed.

Evaluation of isolated acidic charge variants verified that deamidation of LC-Asn-30 in one (83%) or two light chains (58%) does impact, but not completely abrogate, the trastuzumab target binding activity. However, a minor effect of HC-Asp-102 isomerization in one heavy chain (95%) on target binding activity was observed. In addition, bottom-up HDX mass spectrometry analysis of cation-exchange chromatography fractions containing one modified light or heavy chain showed almost no effect of both chemical degradations on trastuzumab higher order structure.

These SPR results are not fully in agreement with a previous reported study on trastuzumab charge variants, in which a pronounced decrease of antiproliferative activity after HC-Asp-102 isomerization was detected in a cell-based assay^25^. To further elucidate the inconsistency of the cell free and the cell-based binding assay results more thorough assessment of mono-/bi-valency and avidity effects is planned in a future independent study.

In summary, none of the assessed degradation products led to a complete loss of target binding activity if only one light or heavy chain of the native trastuzumab was affected. Similar results were also obtained for the deamidation variants of another antibody in clinical development^30^. However, the isomerization of one LC-Asp-92 in a human monoclonal IgG2 has been reported to deactivate both antigen-binding regions^51^. Hence, further antibody-specific in vivo studies for determining a full understanding of the biological impacts of specific degradation sites need to be conducted^52^.

Another aim of this study was to compare the extent of relevant (>1%) Asn deamidation, Asp isomerization and Met oxidation in the conserved IgG Fc region of manufactured trastuzumab with those from endogenous human serum IgG levels from healthy donors. Chelius et al.^32^ identified the conserved HC-Asn-387/392/393 motive as the most susceptible deamidation site of recombinant IgG1 mAbs. However, no vulnerable Asp residue was identified in an accelerated temperature study with trastuzumab^28^.

The observed Asn deamidation, Asp isomerization and Met oxidation content for trastuzumab reference material of 1–4% relative abundance at reduced peptide level is in agreement with previous studies on IgG1 chemical degradations^28,30,32,38,41^.

For the susceptible deamidation motive, HC-Asn-387/392/393 and the two Met oxidation sites HC-Met-255 and HC-Met-431, we found 5- to 10-fold elevated values for endogenous human IgG pools (raised from 1–3% to up to 20%, 16% and 10%, respectively), whereas the degradation content for the non-prone Fc residues (HC-Asp-283, HC-Asn-364, and HC-Asp-404) was comparable to the non-stressed trastuzumab reference material. The detected endogenous human Fc Asn deamidation levels are significantly higher than those observed for liquid drug formulation stored at 5 °C and comparable to in vitro PBS incubation and in vivo administration experiments (Fc deamidation at HC-Asn-387/392/393 of around 10% at day 15).

Emerging biomedical applications of mass spectrometry-based approaches for the in vitro and in vivo assessment of chemical antibody modifications have been reported^50,53–58^.

In the study presented here, we applied in vitro and in vivo stress conditions combined with Protein A and ion exchange chromatography, proteolytic peptide mapping, quantitative LC–MS, HDX-MS, and evaluation of target binding activity by SPR to identify and assess the physiological degradation of chemical modification sites in the CDRs and conserved Fc region of trastuzumab. The reported methodologies and results may also be relevant for other major classes of biopharmaceuticals such as Fc-fusion proteins, protein scaffolds, and bispecific antibodies^59–61^.

In this study, with the model mAb, trastuzumab, we show that the degradation of the susceptible residues located in the CDR (Asn-30, Asp-102, and Asn-55) and the conserved Fc (Asn-387/392/393, Met-255, and Met-431) were formed in vivo with significantly higher abundance and rate (within days) compared to that observed for bio-process and real-time storage conditions. This raises the question whether current critical quality attribute approaches^62,63^, which focus on assessment of low level alterations in chemical modifications (single digit % abundance range) in formulation, realistically add value to maintaining product quality from a patient’s safety perspective.

## Methods

### Monoclonal antibody rhuMAb HER2

The humanized monoclonal IgG1 antibody trastuzumab was expressed in a Chinese hamster ovary cell system. The antibody was manufactured at Roche Diagnostics GmbH, Penzberg, Germany. Trastuzumab was formulated at a concentration of 25 mg/mL in a Histidine-HCl buffer system (60 mM) at pH 6.

### In vitro incubation of mAb in PBS and SCID beige mouse serum

To simulate antibody Asn deamidation and Asp isomerization in vivo, the recombinant IgG1 antibody was spiked up to a final concentration of 1 mg/mL in PBS (pH 7.4) and SCID beige mouse serum at 37 °C. The solutions were incubated and sampled over time (0, 1, 7, 12, and 15 days). 0.1% sodium azide was added to prevent bacterial growth. Following incubation the samples were purified by Protein A chromatography and thereafter stored at −80 °C for further analysis.

### In vivo administration of mAb, sample collection, and preparation

Female SCID beige mice, at an age ranging from 7 to 9 weeks, were obtained from Charles River Laboratories Inc., Sulzfeld, Germany. The study was conducted in accordance with the German animal welfare law and approved by the local governmental animal ethics committee (Regierungspraesidium Oberbayern, Bavaria, Germany). A single dose of trastuzumab was injected intravenously (IV) via the lateral tail vein at a dose level of 50 mg/kg. At different time points (1, 7, 12, and 15 days) blood was collected via the retro bulbar venous plexus under light anesthesia with isoflurane, until total bleeding. Serum was obtained by centrifugation of the blood through Microvette® 500 Z-Gel tubes (3 min, 9000×*g*). Serum from three non-treated animals was also prepared to serve as blank matrix. The serum samples were directly purified by Protein A chromatography and stored at −80 °C until further analysis. To achieve sufficient amounts of trastuzumab for all characterization methods required, serum from 5 to 15 animals per time point were pooled.

### Isolation of mAb from in vitro and in vivo time-course studies

Trastuzumab was isolated from PBS and mouse serum by Protein A affinity chromatography. Protein A purification was carried out on an Agilent Bravo robotic system (AssayMAP 96 AM head) using Protein A AssayMAP cartridges. Samples were loaded (2 μL/min) onto Protein A cartridges following equilibration (PBS; one column volume). In order to wash off all unbound molecules, the cartridges were cleaned with six column volumes PBS at a flow rate of 25 μL/min with 200 μL washing buffer. Samples were then eluted with 50 μL elution buffer (12 mM HCl, 0.1 M NaCl, pH 2) at a flow rate of 3 μL/min. Following elution, the solution was immediately adjusted to neutral pH by addition of 40 mM Histidine Buffer (pH 6.5). Samples were either directly stored at −80 °C, or first concentrated (SpeedVac, 25 °C) to give a final concentration of at least 0.4 mg/mL for subsequent analytical experiments.

### Isolation of endogenous human IgG by Protein G purification

Human sera were obtained from healthy volunteers aged between 30 and 50 years. Healthy donors were recruited at the University of Heidelberg (Germany), after giving their written informed consent. The protein G purified serum IgG was obtained from a clinical study approved by the appropriate local Ethics Committee and Institutional Review Board of the University of Heidelberg (S-040). 100 µL-aliquots were diluted with 900 µL PBS and applied on a HiTrap Protein G HP column (1 mL) using an ÄKTA purifier 10 system (GE Healthcare, Munich Germany) at 4 °C and a flow rate of 0.5 mL/min. After washing with 10 mL PBS, pure IgG fractions were eluted with 100 mM glycine pH 2.6 and immediately neutralized with 1.5 mM Tris-HCl (pH 8.8). Purity of the obtained samples was checked by SDS electrophoresis and Coomassie staining.

### Proteolytic digest of mAb time-course studies in PBS and mouse serum

The trastuzumab samples were first denatured in 0.2 M His-HCl, 8 M Gua, pH 6 by diluting 50 µL purified mAb sample in a total volume of 100 µL. For reduction, 2 µL of 0.1 g/mL dithiothreitol (DTT) was added followed by incubation at 50 °C for 1 h. Thereafter the buffer was exchanged to a digestion buffer (0.02 M His-HCl, pH 6) using Zeba™ spin desalting columns (7 K MWCO, 0.5 mL, Thermo Scientific™). Subsequently 2 µL of a 0.25 mg/mL trypsin solution (Trypsin Proteomics grade, Roche, Penzberg, Germany) in 10 mM HCl was then added to each eluate and the solutions incubated at 37 °C for 18 h ± 2 h.

### Proteolytic digest of endogenous, human IgGs

For the detection and relative quantification of Asn deamidation, Asp isomerization and Met oxidation of human IgGs at peptide level, the mAbs were first denatured in 0.2 M His-HCl, 8 M Gua at pH 6 by diluting 100 µg of mAb to a total volume of 300 µL. For reduction, 10 µL of 0.1 g/mL DTT was added followed by incubation at 50 °C for 1 h. Buffer exchange to digestion buffer (20 mM His-HCl, pH 6) was then performed using NAP-5^™^ gel filtration columns (GE Healthcare Life Sciences). The eluates (500 µL) were subsequently mixed with 10 µL of a solution of 0.25 mg/mL trypsin (Trypsin Proteomics grade, Roche, Penzberg, Germany) in 10 mM HCl and incubated at 37 °C for 18 ± 2 h.

### Analysis of proteolytic peptides of in vitro and in vivo time-course studies by liquid chromatography mass spectrometry (LC–MS)

The tryptic peptide mixture (~3.5 µg) was separated by RP-UPLC (ACQUITY, Waters, Manchester, UK) on a C18 column (BEH C18 1.7 µm, 2.1 × 150 mm; Waters, Manchester, UK) and the eluate analyzed online with a SynaptG2 QTOF electrospray mass spectrometer (Waters). The mobile phases consisted of 0.1% formic acid in water (solvent A) and 0.1% formic acid in acetonitrile (solvent B). The chromatography was carried out using a gradient from 1% to 35% solvent B in 45 min using a flow rate of 0.3 mL/min and temperature of 65 °C. UV absorption was monitored at a wavelength of 220 nm. Data acquisition was controlled by the MassLynx and Acquity UPLC software packages (Waters GmbH). Parameters for MS detection were adjusted according to general experience available from peptide analysis of recombinant antibodies.

### Analysis and identification of proteolytic peptides in human IgG by liquid chromatography tandem mass spectrometry (LC–MS/MS)

Chromatographic separation of tryptic peptides (~3.5 µg) was carried out on an UltiMate3000 Rapid Separation system (U3000 RSLC, Thermo Fisher Scientific, Germering, Germany) using a C18 column (BEH C18 1.7 µm, 2.1 × 150 mm; Waters, Manchester, UK) and the eluate online analyzed with an Orbitrap Fusion electrospray mass spectrometer (Thermo Fisher Scientific, Bremen, Germany). The mobile phases consisted of 0.1% formic acid in water (solvent A) and 0.1% formic acid in acetonitrile (solvent B). The chromatography was carried out using a gradient from 1 to 40% solvent B in 110 min at a flow rate of 0.3 mL/min and temperature of 65 °C. UV absorption was monitored at a wavelength of 220 nm.

Data parameters for MS and MS/MS detection were adjusted according to general experience available from peptide analysis of recombinant antibodies. MS/MS experiments were performed on-line on an Orbitrap Fusion instrument (Thermo) performing a full scan acquisition (in the Orbitrap), followed by a MS/MS scan of the top five most intense ions of each full scan (in the Ion Trap) using helium as collision gas (low-energy CID). The collision energy was adjusted according to stability and mass of the parent ion. MS/MS-data were analyzed manually using PEAKS software for mass detection and data interpretation.

### Data analysis for the relative quantification of deamidation, isomerization, and oxidation levels

Peptides of interest were identified by manual search of the *m*/*z*-values within the experimental mass spectrum. For the quantification, specific ion current (SIC) chromatograms of peptides of interest were generated on the basis of their monoisotopic mass, detected charge states, and isotopes using an in-house developed module in the GRAMS AI software (Thermo Scientific, V8.0). Relative amounts of Asn deamidation, Asp isomerization, and Met oxidation were calculated by manual integration of modified and unmodified peptide peaks. The varying ionization efficiencies of the different peptide moieties were not taken into account and hence only relative quantification values are reported.

### Charge variant analysis by cation-exchange chromatography

Initial characterization of IgG charge variants was performed by cation-exchange chromatography (CEC) using a ProPac WCX-10 analytical cation-exchange column (4 × 250 mm; Dionex Softron GmbH) on an UltiMate3000 HPLC system (Dionex Softron GmbH). Separation was achieved with a gradient from 15% to 55% Eluent B in 30 min, 5 min at 55%, 55% to 100% in 1 min and 8 min at 100% Eluent B (Eluent A: 0.1 M NaCl; Eluent B: 10 mM Na_2_HPO_4_/NaH_2_PO_4_ × H_2_O, pH 7.5). A flow rate of 0.8 mL/min and column temperature and pressure of 25 °C and 120 bar were applied. The UV absorption was monitored at 214 nm. Samples (~20 µg; in vivo samples from day 7–15: 250 µL) were pre-treated with 1% CpB (1 mg/mL, Roche Diagnostics GmbH) for 20 min at 37 °C and injected for chromatographic analysis.

### Fractionation of charge variants by preparative cation-exchange chromatography

In addition, CEC fractionation was performed to collect mAb charge variants, for characterization purposes, using a Source 15 S cation-exchange column (0.8 × 25 cm, GE Healthcare). A linear gradient from 0% to 35% Eluent B (25 mM Tris-HCl, 100 mM NaCl, pH 7.4) at a flow rate of 0.7 mL/min (at room temperature) was applied. Chromatographic separation of 60 mg mAb was performed on an Äkta Explorer 100 equipped with UV detection at 280 nm and a Fra-950 fraction collector (GE Healthcare). The specific peaks were collected in 1 mL fractions. The collected fractions were first concentrated by Amicon Ultra 10 kDa filter devices (Millipore), followed by buffer exchange to 25 mM Tris-HCl, 10 mM NaCl, pH 7.4 using PD10 columns (GE Healthcare). The purity of the collected fractions was verified by analytical SEC and analytical CEC.

### Size variant analysis by size-exclusion chromatography

SEC was carried out using a TSK-Gel G3000 SWXL column (7.8 × 300 mm, 5 µm particle size; Tosoh Bioscience). An isocratic elution using 100% running buffer (200 mM KH_2_PO_4_/K_2_HPO_4_, 250 mM KCl, pH 7) at a flow rate of 0.5 mL/min was used for chromatographic separation on an UltiMate3000 HPLC system (Dionex Softron GmbH) equipped with UV detection at 280 nm. 12 µg of each in vitro sample (25 µg of each IEC fraction) were injected for the chromatographic analysis (at room temperature). Relative quantification was performed by manual integration and comparison of peak areas.

### Analysis of target binding by surface plasmon resonance for in vitro and in vivo time-course studies and preparative fractions

The interaction between in vitro and in vivo trastuzumab time-course samples with the specific target protein was measured by SPR using a Biacore T200 instrument (GE Healthcare). The specific target protein was immobilized onto an activated Biacore C1-biosensor chip (GE Healthcare) via amine coupling to reach a high coupling density of more than 1000 RU. This provides a high probability of avide binding of both antibody valencies. The assay was carried out at room temperature with HBS-EP + buffer (GE Healthcare) as running and dilution buffer. Samples of ~2 nM of mAb were injected at a flow rate of 10 µL/min for 90 s, followed by a 60 s dissociation phase (10 µL/min). Regeneration of the chip surface was achieved by injection of 3 M magnesium chloride twice for 45 s and then 30 s at a flow rate of 10 µL/min. The concentration of each sample was checked and corrected for, using a Protein A affinity assay in parallel. The activity of the starting material was set to 100% (0 days incubation of in vitro time-course was set to 100%, 1 day in vivo time-course was set to 100% activity) and the data normalized accordingly.

### SDS-polyacrylamide gel electrophoresis

Proteins were separated by sodium dodecyl sulfate polyacrylamide gel electrophoresis (SDS-PAGE) under denaturing conditions. For this purpose a NuPAGE 4–12% Bis-Tris Gels was used. Prior to loading, the protein samples were incubated in 1×NuPAGE LDS Sample Buffer (non-reducing) at 70 °C for 10 min. After incubation, 20 µL of each sample (~ 2 µg) were loaded into the gel wells. Electrophoresis was carried out in X cell sure lock systems at 120 mA (200 V, 25 W) for 35 min in 1×NuPAGE MES SDS Running Buffer. Subsequently the gels were washed three times in 100 mL ultrapure water for 10 min at 3 rpm. The protein bands were stained in 100 mL SimplyBlue SafeStain with constant shaking at 3 rpm for 60 min. Unbound dye was removed by washing the gels in 100 mL ultrapure water over night (3 rpm). The molecular weight of each protein band was determined using the standard protein marker Mark12 unstained standard, range 200–2.5 kDa.

### Hydrogen/deuterium exchange-mass spectrometry (HDX-MS)

*Solutions and Buffers*: Working solutions of the antibody samples were prepared at a concentration of 6.6 mg/mL with equilibration buffer (5 mM KH_2_PO_4_, 5 mM K_2_HPO_4_, pH 7.4), following concentration by centrifugation at 10,000 rpm using Vivaspin® 500 ultrafiltration spin columns (10,000 MWCO PES, Sartorius Stedium Biotech GmbH).

Deuterium labeling was achieved by 1 in 20 dilution of the samples with the deuterium-containing labeling buffer (5 mM K_2_HPO_4_, 5 mM KH_2_PO_4_/D_2_O-Puffer, pD 7) at room temperature. Quenching of the deuterium uptake was performed by 1 in 2 dilution of the labeled sample with ice-cold quenching buffer (50 mM K_2_HPO_4_, 50 mM KH_2_PO_4_, 500 mM TCEP and 4 M guanidine, pH 2.35) resulting in a final pH of 2.6. This resulted in 55 pmol mAbX on column for each sample injection.

*HDX-MS time survey*: A kinetic experiment comparing the isolated isomerized species LC-Asn30-deam (A5) and HC-Asp102-deam (B1) with the Main peak (non-degraded, native trastuzumab), IEC fractions A5, B1, and Main, respectively, was performed with eight sampling points over a 4 h time course, namely: 0, 0.25, 1, 10, 30, 60, 150, and 240 min. All samples were shock frozen on dry ice following quenching and stored at −80 °C. LC–MS measurement was performed over the following 3 days. The non-deuterated (*t* = 0) controls were measured in triplicate, while all other time points were single-fold experiments only.

*LC/MS measurement:* The quenched deuterated protein samples were online digested and analyzed with a nanoAcquity UPLC SynaptG2 HDMS QTOF mass spectrometric system (Waters Corp.) equipped with temperature controlled column chambers for online pepsin digestion (15 °C) and UHPLC separation (0 °C), and further processed as described previously ^42^.

### Data availability

All data generated or analyzed in this study are included in this published article (and its Supplementary Information files). Raw data were generated at the Roche Diagnostics GmbH, Penzberg, Germany large-scale facility for therapeutic protein production and are permanently store in the Electronic Record Management System (ERMS). Derived raw and explanatory data supporting the findings of this study are available from the corresponding author upon request.

## Electronic supplementary material

Supplementary Information
