# Supplementary material for: Assessment of susceptible chemical modification sites of trastuzumab and endogenous human immunoglobulins at physiological conditions
Source: Commun Biol. 2018 Apr 5;1:28. doi: 10.1038/s42003-018-0032-8 (PMC6123738; doi:10.1038/s42003-018-0032-8)
Supplement: Supplementary file 1 — Supplementary Information [file 42003_2018_32_MOESM1_ESM.pdf]

(A)

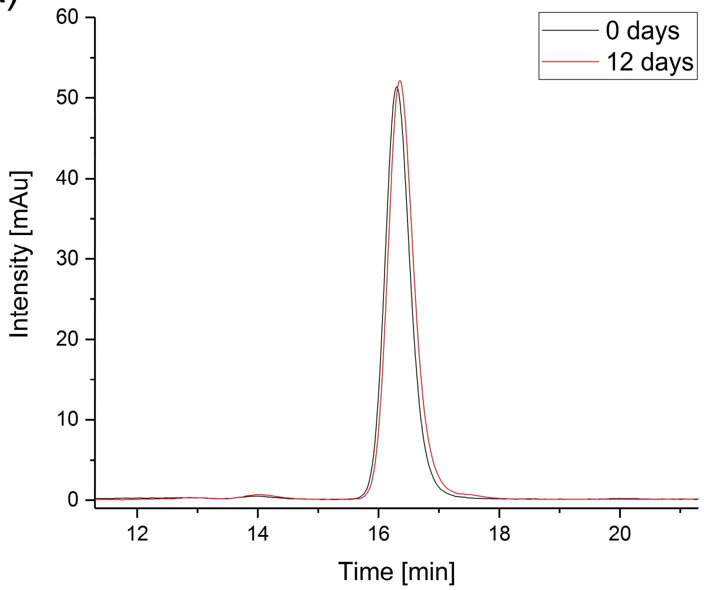

0 days
12 days
Intensity [mAu]
Time [min]
60
50
40
30
20
10
0
12
14
16
18
20

(B)

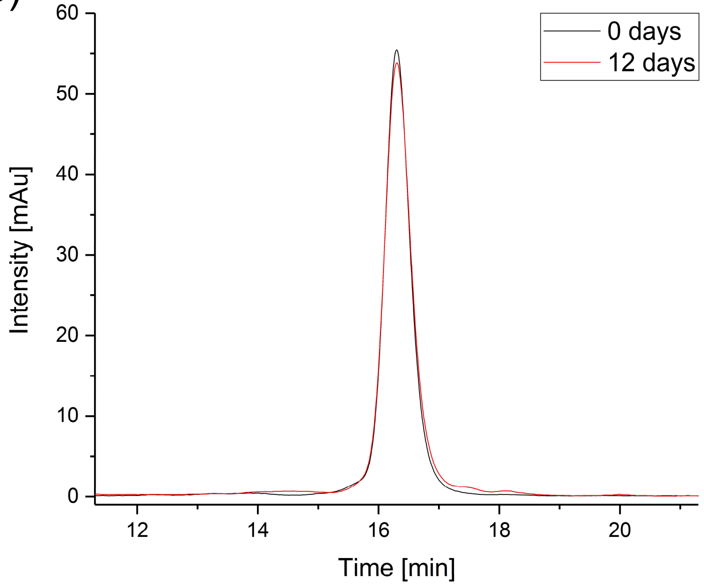

0 days
12 days
Intensity [mAu]
Time [min]
60
50
40
30
20
10
0
12
14
16
18
20

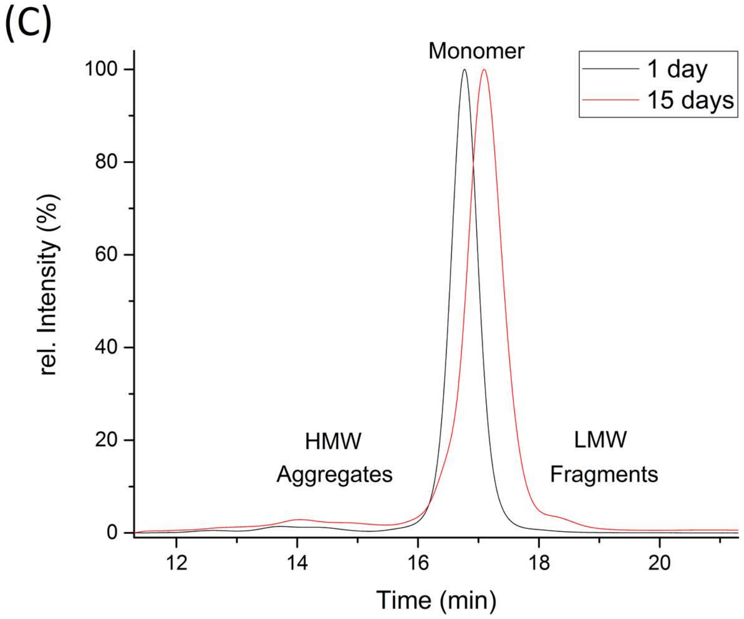

Supplementary Figure 1

Size exclusion chromatography (SEC) of trastuzumab reference material subjected to (A) PBS incubation, (B) serum incubation and (C) *in vivo* administration: The SEC analysis of the sample day 1 and 15 (Figure 1C) were performed in two separate analyses at different days. The monomer peaks of both samples (day 1 and 15) eluted at the retention time of the corresponding reference material.

Fraction A5 – Light chain peptic peptides

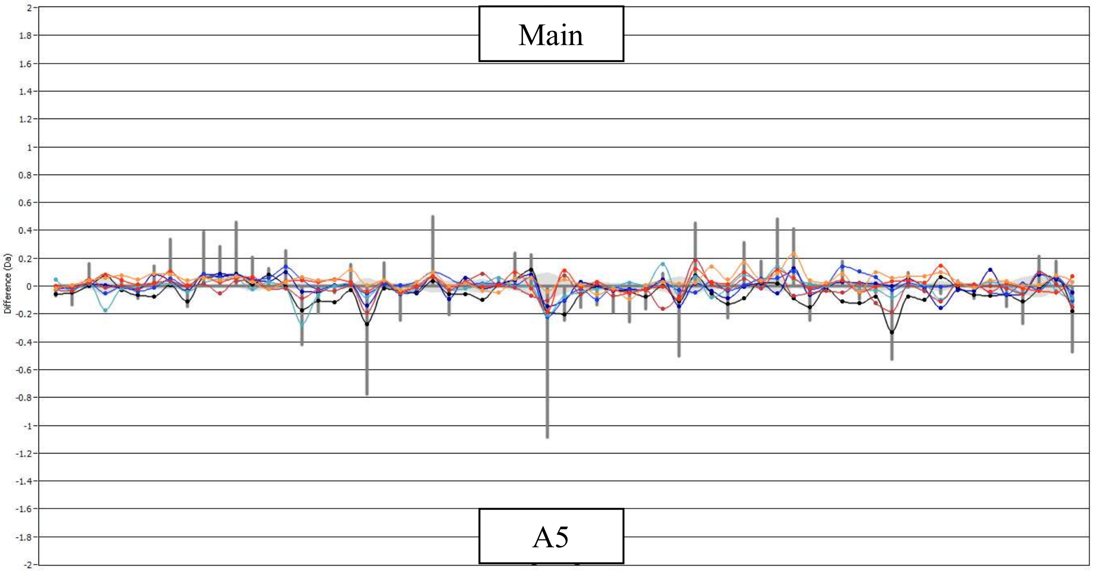

Fraction B1 – Heavy chain peptic peptides

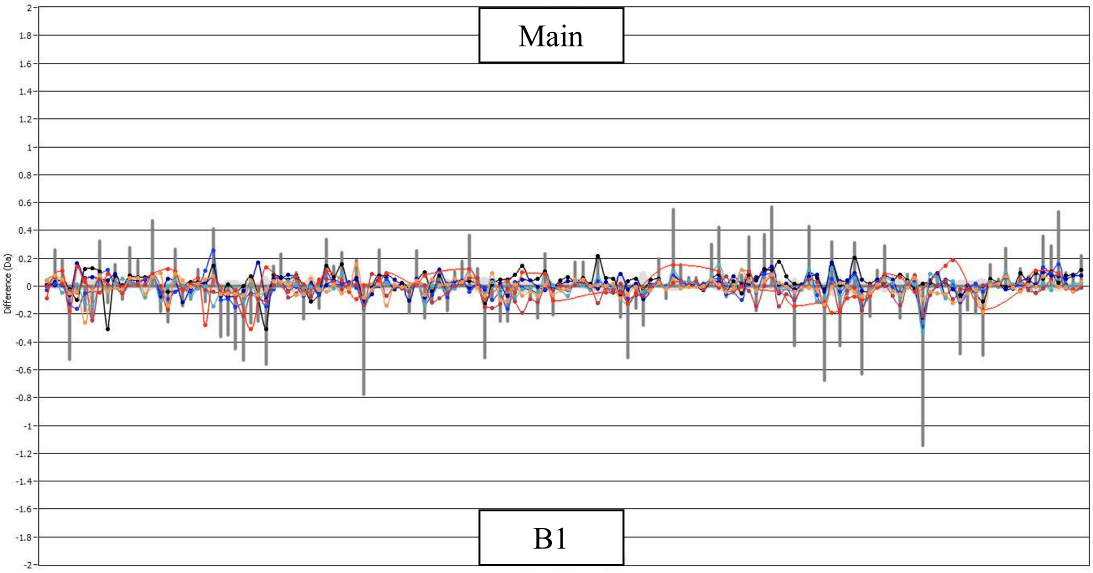

Supplementary Figure 2

HDX-MS Difference Index Butterfly Plot for the LC and HC peptic peptides of the trastuzumab main IEC fraction versus the acidic fraction A5 and basic fraction B1, respectively, showing the deuterium uptake differences (Da) at the seven measured deuteration time points (min.): Orange 0.5, Red 1.0, Purple 10, Aqua 30, Blue 60, Navy 150 and Black 240, and summed as grey sticks.

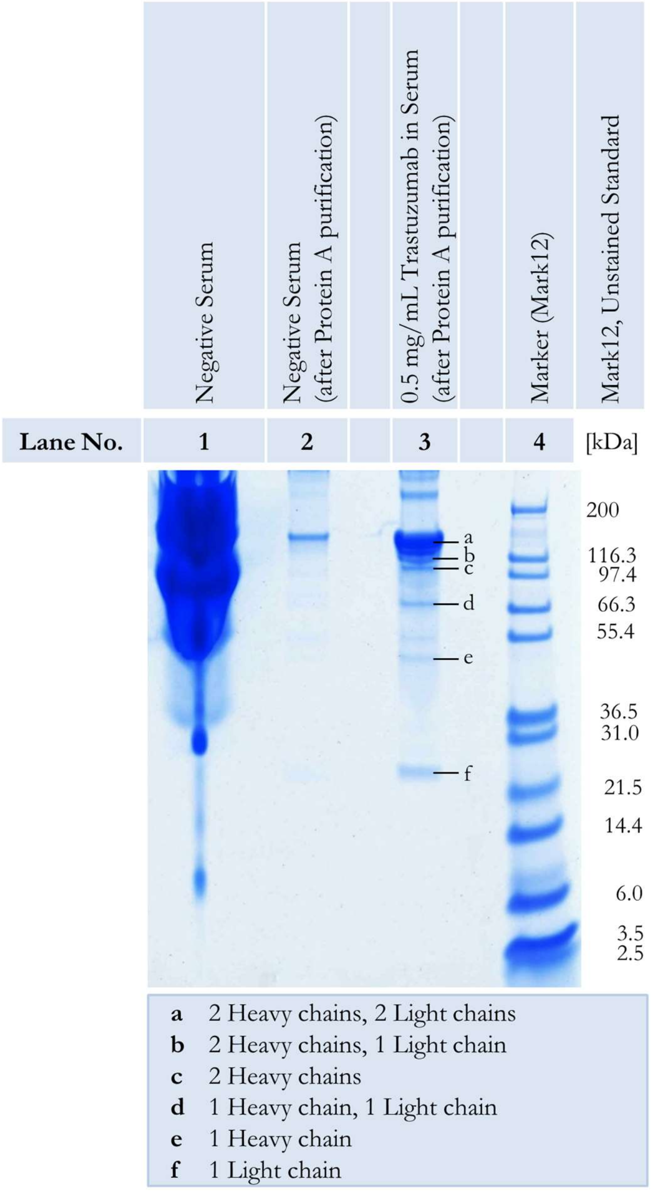

Supplementary Figure 3

SDS-PAGE analysis of trastuzumab purified from SCID *beige* mouse serum by Protein A chromatography.

(A)

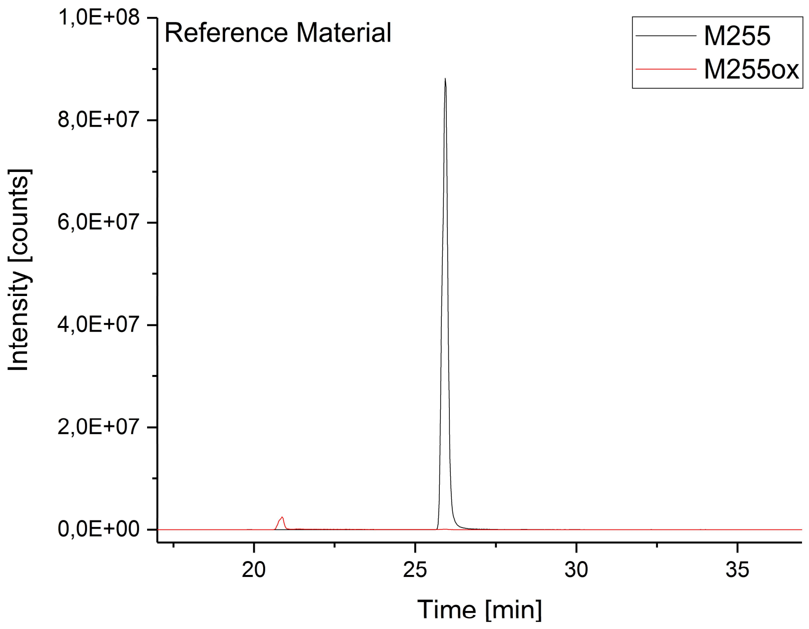
Reference Material
M255
M255ox
1,0E+08
8,0E+07
6,0E+07
4,0E+07
2,0E+07
0,0E+00
Intensity [counts]
20
25
30
35
Time [min]

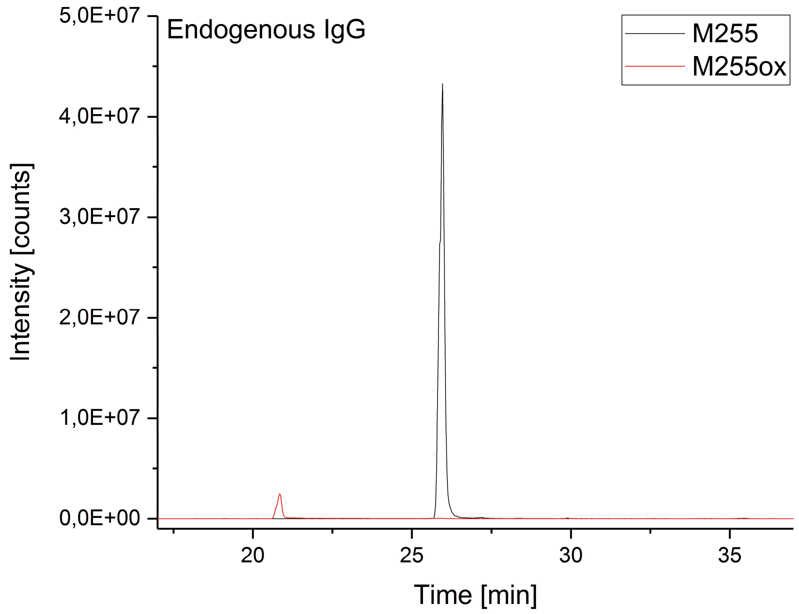
Endogenous IgG
M255
M255ox
5,0E+07
4,0E+07
3,0E+07
2,0E+07
1,0E+07
0,0E+00
Intensity [counts]
20
25
30
35
Time [min]

(B)

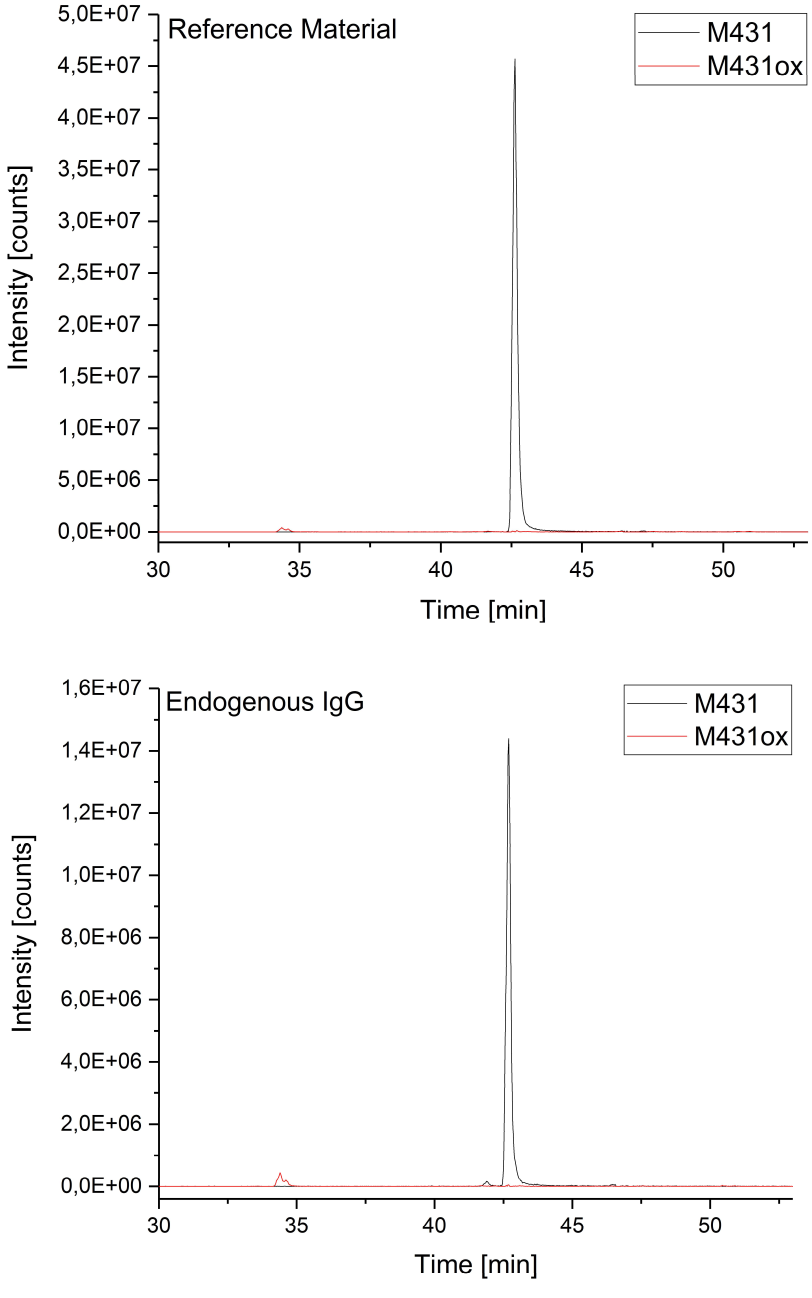
Reference Material
M431
M431ox
Intensity [counts]
5,0E+07
4,5E+07
4,0E+07
3,5E+07
3,0E+07
2,5E+07
2,0E+07
1,5E+07
1,0E+07
5,0E+06
0,0E+00
30
35
40
45
50
Time [min]
Endogenous IgG
M431
M431ox
Intensity [counts]
1,6E+07
1,4E+07
1,2E+07
1,0E+07
8,0E+06
6,0E+06
4,0E+06
2,0E+06
0,0E+00
30
35
40
45
50
Time [min]

(C)

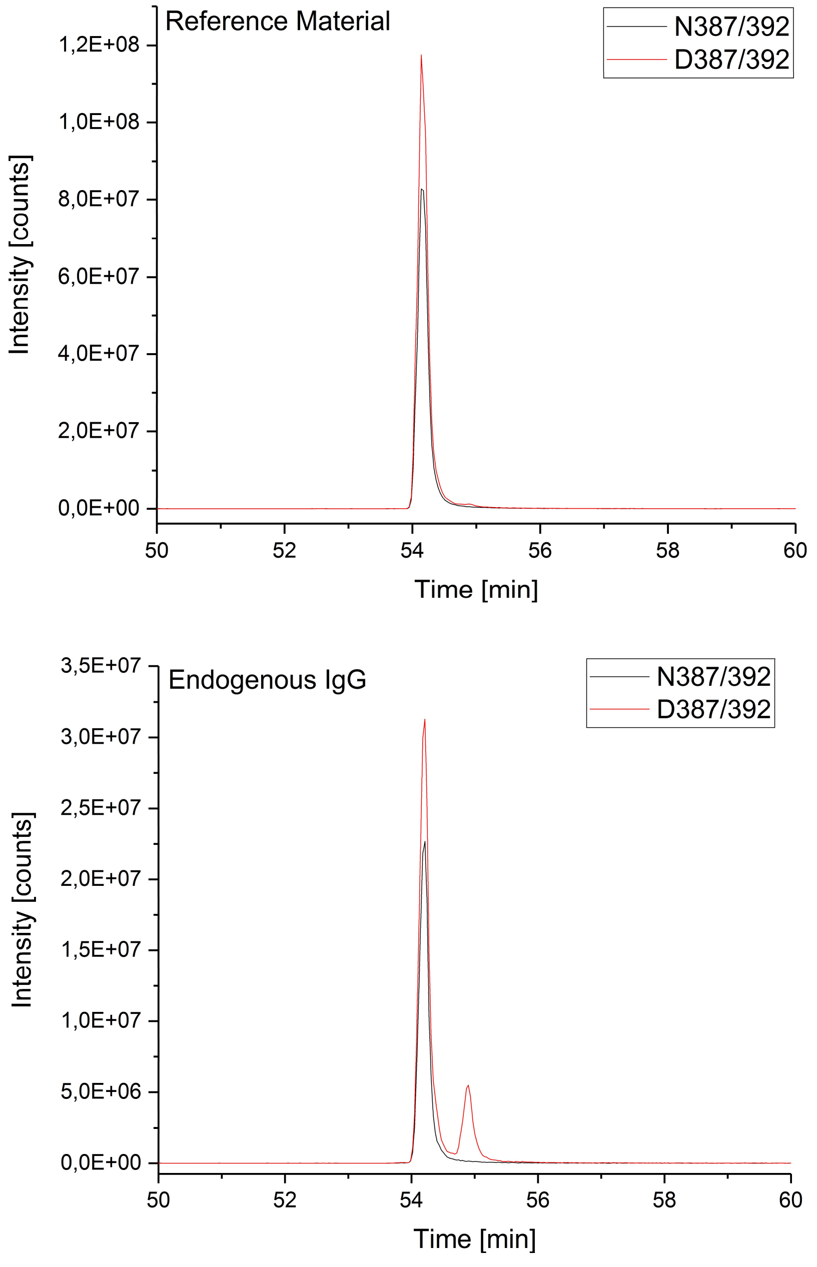

Supplementary Figure 4

Extracted ion chromatograms of trastuzumab reference material and endogenous human IgG peptides containing (A) HC-Met(M)-255, (B) HC-Met(M)-431, (C) HC-Asn(N)-387/392, and their corresponding chemical oxidation/deamidation (red line). Quantification results are summarized in Table 4.
